# MicroRNA profile of polyunsaturated fatty acid treated glioma cells reveal apoptosis-specific expression changes

**DOI:** 10.1186/1476-511X-10-173

**Published:** 2011-09-30

**Authors:** Nóra Faragó, Liliána Z Fehér, Klára Kitajka, Undurti N Das, László G Puskás

**Affiliations:** 1 Functional Genomics Laboratory, Biological Research Center of the Hungarian Academy of Sciences, Temesvári krt.62, Szeged H-6726, Hungary; 2 Avidin Ltd., Közép fasor 52, Szeged H-6726, Hungary; 3 UND Life Sciences, 13800 Fairhill Road, #321, Shaker Heights, OH 44120, USA; 4 Jawaharlal Nehru Technological University, Kakinada-533 003, India; 5 Krishna Institute of Medical Sciences, Secunderabad-500 003, India; 6 Bio-Science Research Laboratory, Gayatri Vidya Parishad College of Engineering, Visakhapatnam-530 048, India

**Keywords:** PUFA, micro RNA, glioblastoma, apoptosis

## Abstract

**Background:**

Polyunsaturated fatty acids (PUFAs) such as γ-linolenic acid (GLA), arachidonic acid (AA) and docosahexaenoic acid (DHA) have cytotoxic action on glioma cells.

**Results:**

We evaluated the cytotoxic action of GLA, AA and DHA on glioma cells with specific reference to the expression of miRNAs. Relative expression of miRNAs were assessed by using high throughput nanocapillary real-time PCR. Most of the miRNA target genes that showed altered expression could be classified as apoptotic genes and were up-regulated by PUFA or temozolomide treatment, while similar treatments resulted in repression of the corresponding mRNAs, such as *cox2*, *irs1*, *irs2*, *ccnd1*, *itgb3*, *bcl2*, *sirt1*, *tp53inp1 *and *k-ras*.

**Conclusions:**

*Our *results highlight involvement of miRNAs in the induction of apoptosis in glioma cells by fatty acids and temozolomide.

## Background

Malignant gliomas are among the most devastating of cancers and are a major cause of mortality in a young population with a median survival time of 9 months following cytoreductive surgery, radiotherapy and chemotherapy. Despite advances in surgery, chemotherapy, and radiotherapy, the prognosis of patients with this fatal disease has not improved significantly over the past 20 years [1,2]. Our previous studies showed that certain polyunsaturated fatty acids, especially γ-linolenic acid (GLA), arachidonic acid (AA), eicosapentaenoic acid (EPA) and docosahexaenoic acid (DHA) have tumoricidal action against glioma cells both in vitro and in vivo [3-7]. Understanding how the signaling pathways involved in surviving and inducing cell death of different glioma cells are regulated during PUFA treatment is important for the development of more effective tumor therapies including PUFAs alone or in combination with other drugs [8]. Several research groups have analyzed the mRNA expression profile of different cancer types to reveal novel gene markers for diagnosis and theraphy and to better understand the regulatory pathways and genetic networks [9,10].

The mechanisms of action that are involved in the effects of PUFAs on cell regulation are complex. Thus, the use of microarrays, which allows the detection of differentially regulated mRNAs of thousands of different genes simultaneously, is a useful tool to elucidate such complex systems. To date, several studies have used microarrays to study the effects of PUFAs on cell regulation, including their effects on brain function [9,10] and cancer [11,12], but only limited data is available as to how PUFAs can modulate the expression of microRNAs [12]. It is now possible to analyze portions of the miRNAome using microarray or high throughput polymerase chain reaction (PCR) methodologies [13,14]. But, to date, no data is available as to the effect of different PUFAs on miRNA expression in glioblastoma cells.

MicroRNAs are small non-coding RNA molecules that are currently being recognized as endogenous physiological regulators of gene expression. These small RNAs are capable of controlling gene expression by repression of translation/transcription ("RNA interference") [15,16]. While different tumor types present specific microRNA signatures, several microRNAs are deregulated in several different tumor entities, suggesting their involvement in the basic processes of tumorigenesis. MicroRNAs may act as oncogenes by inhibiting translation of tumor suppressor mRNAs, but they can act as tumor suppressor genes as well, by inhibiting

translation of oncogenic mRNAs [16-18]. MicroRNAs may also interfere with other characteristics of the malignant phenotype, including chemoresistancy, angiogenesis, invasion, metastasis or immunogenicity [18-20]. However, how miRNAs affect the drug sensitivity of cancer cells is still unknown. Here we report miRNA profiling study of three different glioma cells treated with three different PUFAs (GLA, AA and DHA) and temozolomide as a chemotherapeutic agent currently used in glioma treatment.

## Materials and methods

### Treatment and cultivation of glioblastoma cell lines

Glioblastoma cells (U373, GBM2, GBM5) were grown at 37°C under 5% of CO2 and 100% humidity in DMEM and RPMI medium supplemented with 10% FCS (Sigma-Aldrich), and penicillin-streptomycin antibiotics. The cells were treated with 50 and 100 μM AA, DHA, TMZ, 75 and 150 μM GLA for 24 hours and then harvested with trypsin and washed with PBS.

### Purification of microRNA from glioblastoma cells

Purification of microRNA was done with the High Pure miRNA Isolation Kit (Roche, Cat. no. 05080576001) as described previously with slight modifications [21]. Briefly: after harvesting, glioblastoma cells (5 × 10^6^) were washed with PBS, collected by centrifugation and lyzed with 400 μl 20% Binding Buffer. 320 μl Binding Enhancer (Roche) was added, mixed and loaded onto the filter columns (Roche). Next the filters were washed in two steps with 500 and 300 μl of Washing Buffer (Roche), then the RNA was eluted by adding 100 μl Elution Buffer (Roche). All other steps were done according to the manufacturer's recommendations. The quality and quantity was assessed spectrophotometrically (Nanodrop, USA) and with 2100 Bioanalyzer (Agilent).

### Profiling of microRNAs by high-throughput, nanocapillary QRT-PCR

Amplification of the samples was followed in real time with an OpenArray NT Cycler (BioTrove Inc., Woburn, MA, now Life Technologies, Applied Biosystems). An aliquot of each Taqman miRNA assay was sent to BioTrove (Woburn, MA) for loading in their OpenArray plates. Taqman assays are loaded by BioTrove in a customer-specified layout. A third fluorescent dye (ROX), present in the Taqman assay mixture, was imaged to provide quality assessment of manufacturing and loading of the arrays.

cDNA samples (or water for no template controls) were added to a 384-well plate containing universal Taqman master mix (Applied BioSystems, P/N 4324018) for OpenArray amplification. The OpenArray autoloader transfers the cDNA/master mix from the plate to the array through-holes by capillary action. Each subarray was loaded with 5.0 μl of master mix containing 1.2 μl of reverse transcribed miRNA. The array is manually transferred to the OpenArray slide case and sealed. The plates were cycled in the OpenArrayNT cycler (up to three arrays simultaneously) under the following conditions: 50°C for 15 seconds, 91°C for 10 minutes, followed by 50 cycles of 54°C for 170 seconds and 92°C for 45 seconds.

The Biotrove OpenArrayNT Cycler System software (version 1.0.2) uses a proprietary calling algorithm that estimates the quality of each individual threshold cycle (*C_T_*) value by calculating a *C_T _*confidence value for the amplification reaction. In our assay, *C_T _*values with *C_T _*confidence values below 300 (average *C_T _*confidence of the non-target amplification reactions plus 3 standard deviations) were considered background signals. Higher *C_T _*confidence levels were considered positive and were analyzed further. Normalization was performed by using median expression of all miRNAs analyzed in the study for each sample.

### Determination of mRNA levels by using QRT-PCR

Total RNA was used for QRT-PCR analysis as described earlier [22]. Briefly, 2 μg of total RNA from each sample were reverse transcribed in the presence of random primers in a total volume of 20 μl. After dilution with 20 μl of water, 1 μl of the diluted reaction mix was used as template in QRT-PCR. The 20 μl reaction volume contained 0.2 mM of dNTP, 1× PCR reaction buffer (ABGene, Epsom, UK), 6 mM of each primer, 4 mM of MgCl_2_, 1× SYBR Green I (Molecular Probes, Eugene, OR) at final concentration, and 0.5 U of thermostart Taq DNA polymerase (ABGene). Amplification was carried out with the following cycling parameters: 600 s heat start at 95°C, 45 cycles of denaturation at 95°C for 25 s, annealing at 60°C for 25 s, and fluorescence detection at 72°C for 15 s. A total of 45 cycles were run. All the PCRs were performed in triplicate. After amplification, a melting curve was created to verify the specificity of the PCR reactions. Relative expression ratios were normalized to GAPDH and HPRT, as widely used housekeeping genes [22]. The PCR primers used in this study are listed in Table S1 (see Additional file 1, Table S1).

### Confirmation of HTS QRT-PCR miRNA expression data by traditional QRT-PCR

The reverse transcription reaction was performed with the TaqMan^® ^MicroRNA Reverse Transcription Kit (Applied Biosystems, United States, California). 350 ng of miRNA from each sample was reverse transcribed in the presence of 5× RT TaqMan^® ^MicroRNA Assays (Applied Biosystems). 8 μl reaction mixture contained 0.2 μl dNTPs, 1.50 μl MultiScribe™ Reverse Transcriptase (50 U/μL), 0.8 μl 10× RT Buffer, 0.9 μl MgCl2, 0.1 μl RNase Inhibitor (20 U/μL), 1.5 μl 5× RT primer and the template in a total volume of 3 μl. Reverse Transcription was carried out with the following cycling parameters in a thermocycler (Bioneer, Daedong, Korea): 16°C for 2 minutes, 42°C for 1 minutes, 50°C for 1 second, 45 cycles, then hold the samples on 85°C for 5 minutes. After dilution with 64 μl of water, 9 μl of the diluted reaction mix was used as template in QRT-PCR.

QRT-PCR was performed on the Excicycler instrument (Bioneer) with the TaqMan protocol. 20 μl PCR mixture contained 10 μl TaqMan^® ^Universal PCR Master Mix (Applied Biosystems), 1 μl of the TaqMan^® ^MicroRNA Assays and 9 μl of the diluted cDNA.

## Results

### MicroRNA profiling of PUFA treated glioblastoma cells

We have analyzed the expression of 112 microRNA by using high-throughput, nanocapillary QRT-PCR (HTS QRT-PCR) (OpenArray system formerly Biotrove, now Applied Biosystems, Life Technologies) in three different human glioblastoma cell lines (U373, GBM3 and GBM5) in triplicates in response to different PUFAs. In this study, we investigated the effects of GLA, DHA and AA that are PUFAs. In order to differentiate specific responses to different PUFAs we analyzed the effect of temozolomide, which is an oral alkylating agent that is used for the treatment of brain tumors [23]. We used temozolomide to identify miRNA expression changes that are due to apoptosis and to see whether there are differences or similarities between the changes caused by PUFAs and temozolomide.

By using nanocapillary QRT-PCR, we generated raw expression data of treated and un-treated control glioblastoma cells. Relative changes in miRNA expression was calculated. Of the 112 miRNAs analyzed, 65 gave significant signals (below Ct value of 26, upon which this technology results in weaker reproducibility with high standard deviation of the repeated runs). 19 miRNAs were selected based on their selective expression changes related to cell lines or PUFA treatment (Table 1). The results for all the analyzed miRNAs are shown in Table S2 where each block represent the relative and average expression of miRNAs of each glioblastoma cells (see Additional file 2, Table S2).

**Table 1:** Results of 19 selected miRNA expression in glioma cells (G2, GBM2; G5, GBM5; U3, U373) in response to different PUFA (A100, A50, arachidonic acid at 100 and 50 μM concentrations, respectively; D100, D50, DHA at 100 and 50 μM concentrations, respectively; G100, G50, GLA at 100 and 50 μM concentrations, respectively) or temozolomide (T100, 100 μM temozolomide) treatment

ASSAY	G2 A100	G2 A50	G2 D100	G2 D50	G2 G100	G2 G50	G2 T100	G5 A100	G5 A50	G5 D100	G5 D50	G5 G100	G5 G50	G5 T100	U3 A100	U3 A50	U3 D100	U3 D50	U3 G100	U3 G50	U3 T100
***hsa-miR-29a***	3,20	4,86	5,43	4,61	n.d.	n.d.	-5,47	-3,88	-3,86	-4,82	-3,19	n.d.	n.d.	-4,27	-2,50	-4,70	n.d.	2,58	-6,85	n.d.	-10,13

***hsa-miR-16***	-0,29	0,51	-0,78	0,22	n.d.	n.d.	-3,78	3,66	2,13	n.d.	n.d.	0,15	-0,22	-3,89	-2,11	-1,80	-1,50	-1,61	-1,83	0,34	-5,82

***hsa-miR-22***	0,01	-0,16	0,57	-0,95	-3,54	-2,27	-3,39	-1,33	0,09	-0,93	0,15	n.d.	n.d.	-3,33	n.d.	n.d.	-0,01	0,13	-0,15	0,22	-1,87

***hsa-miR-20b***	3,58	3,25	-0,41	0,40	n.d.	n.d.	-2,47	-0,56	0,26	-0,04	0,49	-3,25	-1,06	-3,06	0,68	0,73	1,63	2,71	-0,05	0,09	-0,50

***hsa-miR-29c***	0,85	0,89	-4,50	-2,03	-1,75	-2,06	-2,41	-4,51	-3,90	-4,48	-1,90	4,07	n.d.	-2,40	1,50	-0,33	-0,38	0,55	3,59	3,00	-0,09

***hsa-miR-30c***	-2,79	-2,92	-4,09	-4,93	-2,48	-2,07	-2,11	n.d.	n.d.	n.d.	n.d.	0,03	-0,37	-1,69	-2,64	0,43	n.d.	5,99	0,36	-0,79	-0,07

***hsa-miR-17***	-1,29	-1,08	-0,87	-0,78	-1,34	-0,29	-1,28	0,01	0,72	0,80	0,58	-4,07	-4,96	-1,30	n.d.	1,08	n.d.	n.d.	n.d.	2,15	-0,06

***hsa-miR-25***	-2,62	-1,15	-4,06	-1,40	-2,00	-1,22	-1,27	n.d.	n.d.	3,50	3,43	-0,75	n.d.	-1,13	0,73	-0,01	2,62	4,19	-0,94	0,20	0,06

***hsa-miR-206***	0,39	-0,49	-2,29	-0,29	-3,46	-2,37	-1,22	n.d.	n.d.	n.d.	n.d.	1,10	0,08	-0,93	-2,52	-2,23	-1,22	-0,71	0,46	0,36	0,08

***hsa-miR-183***	0,32	-0,84	0,60	0,80	n.d.	4,02	-1,09	n.d.	n.d.	1,38	0,47	0,24	0,69	-0,79	1,37	0,55	1,80	0,90	2,18	1,47	0,40

***hsa-miR-224***	0,27	0,24	-0,92	-0,32	-1,47	-2,41	-0,97	n.d.	-0,14	-2,36	-1,82	-3,83	-1,45	-0,37	0,61	0,14	2,57	2,43	0,59	1,60	0,53

***hsa-miR-145***	-5,70	-4,30	-7,35	-2,37	-2,72	0,26	-0,77	-1,76	-1,50	-1,19	-1,33	-1,78	-1,87	-0,14	0,89	0,73	0,65	0,37	0,95	0,97	1,36

***hsa-miR-26a***	-0,87	-0,09	-0,77	-0,27	-0,36	0,32	-0,57	-1,09	-1,17	-1,86	-0,66	-2,07	-1,01	-0,08	5,66	5,63	1,69	-0,42	3,17	n.d.	2,85

***hsa-miR-181a***	n.d.	n.d.	n.d.	n.d.	1,83	0,75	-0,55	3,51	3,96	3,59	4,30	-0,84	0,28	-0,01	n.d.	2,42	-2,86	0,73	0,75	-0,08	4,01

***hsa-miR-208***	-1,97	-0,79	0,21	-0,55	-1,29	-2,44	-0,46	4,11	3,85	1,18	1,28	-0,64	0,53	0,86	n.d.	n.d.	n.d.	n.d.	n.d.	n.d.	n.d.

***hsa-miR-143***	-1,28	0,33	-1,52	-0,90	0,25	n.d.	-0,37	-3,84	-3,82	-2,15	-1,19	1,51	0,06	1,61	n.d.	n.d.	n.d.	n.d.	n.d.	n.d.	n.d.

***hsa-miR-20a***	0,76	0,59	-0,62	-0,33	-1,90	-1,60	-0,32	n.d.	n.d.	n.d.	n.d.	2,25	1,41	2,20	n.d.	n.d.	n.d.	n.d.	n.d.	n.d.	n.d.

***hsa-miR-149***	0,47	-0,40	-0,06	-0,01	-0,87	-0,60	1,20	0,93	0,08	3,19	0,74	3,80	2,63	4,82	n.d.	n.d.	n.d.	n.d.	n.d.	n.d.	n.d.

***hsa-miR-125b***	0,73	-0,01	0,31	0,67	n.d.	n.d.	n.d.	-2,19	-2,82	-2,35	-1,35	0,01	-0,92	n.d.	n.d.	n.d.	n.d.	n.d.	n.d.	n.d.	n.d.

Based on the results obtained, we could conclude that there are specific expression changes in the miRNA levels of PUFA treated cells. Most of the miRNA expression changes can be dedicated to the general apoptotic cell death (with all PUFAs as well as temozolomide), such as mir-34, mir-25, mir-17, mir-26a, mir-29c, mir-31, mir-200a, mir-206 in the three cell lines tested. The same general effect, up-regulation of mir-140, mir-323 and mir-133b could be seen but only in U373 cells, but not in GBM2 nor in GBM5 cells (Table 1). Besides non-specific modification of miRNA expression, we could detect temozolomide and PUFA treatment-specific alterations in miRNA level. In case of temozolomide we found specific up-regulation of mir-182, and down-regulation of mir-16 and mir-183. Mir-143 was found to be repressed and mir-20b was induced by PUFA treatments (Table 1).

The expression of mir-125b was repressed in GBM5 cells only, while, mir-197 was up-regulated in U373 cells. The same cell-type specificity could be observed in the case of mir-206 that was down-regulated in GBM2 cells, but was up-regulated in U373 cells. These differences in the expression of certain types of miRNAs in response to various PUFAs by different glioma cells could be due to differences in the signalling pathways in different types of tumor cell lines, even though the three studied cell lines were glioblastoma cells.

### Validation of HTS QRT-PCR data by traditional QRT-PCR

However, several studies have already validated relative gene expression data obtained by HTS QRT-PCR miRNA profiling with this technology (24). Therefore, we selected six key miRNAs and determined their relative expression ratios in samples treated with different PUFAs and temozolonide relative to untreated samples in all the three glioma cells. miRNA expression was determined by using Taqman probes (Applied Biosystems, Life Technologies) in both methods used. Data obtained by both HTS QRT-PCR and traditional QRT-PCR resulted in very consistent data, 75% of the data showed similar changes to each other and we obtained only 4% more data with the traditional QRT-PCR. Details of the confirmatory data can be found in Table 2.

**Table 2:** Correlation of relative miRNA expression obtained from HTS QRT-PCR or traditional QRT-PCR.

	**miRNA-16**	**A**	**D**	**G**	**T**			**miRNA-30c**	**A**	**D**	**G**	**T**
				
GBM2	***HTS QRT-PCR***	-0,2	-0,78	n.d.	-3,78		GBM2	***HTS QRT-PCR***	-2,78	-4,08	-2,4	-2,1
	***QRT-PCR***	-1,02	-1,02	-0,5	-1,59			***QRT-PCR***	-5,63	-1,86	-2,16	-1,44
	***confirm***	***n***	***y***		***y***			***confirm***	***y***	***y***	***y***	***y***
GBM5	***HTS QRT-PCR***	n.d.	3,49	-0,75	-1,13		GBM5	***HTS QRT-PCR***	-3,88	-4,8	n.d.	-4,26
	***QRT-PCR***	0,1	0,87	-0,92	-3,32			***QRT-PCR***	-5,84	-2,71	-2,69	-2,72
	***confirm***		***y***	***y***	***y***			***confirm***	***y***	***y***		***y***
U373	***HTS QRT-PCR***	n.d.	-0,02	-0,15	-3,78		U373	***HTS QRT-PCR***	-2,49	n.d.	-6,84	-10,13
	***QRT-PCR***	-1,25	-1,35	-1,66	-1,2			***QRT-PCR***	-4,88	-3,17	-4,78	-3,74
	***confirm***		***n***	***n***	***y***			***confirm***	***y***		***y***	***y***
												
	**miRNA-20b**	**A**	**D**	**G**	**T**			**miRNA-143**	**A**	**D**	**G**	**T**
				
GBM2	***HTS QRT-PCR***	3,58	-0,4	n.d.	-2,47		GBM2	***HTS QRT-PCR***	-1,2	-1,5	2,61	-0,36
	***QRT-PCR***	1,18	-1,02	-0,11	-2,07			***QRT-PCR***	-1,3	-5,38	-3,8	-2,44
	***confirm***	***y***	***y***		***y***			***confirm***	***y***	***y***	***n***	***n***
GBM5	***HTS QRT-PCR***	4,11	1,17	-0,6	0,85		GBM5	***HTS QRT-PCR***	-1,76	-1,18	-1,77	-0,14
	***QRT-PCR***	3,81	4,34	4,02	2,31			***QRT-PCR***	-3,8	-3	-3,62	-1,53
	***confirm***	***y***	***y***	***n***	***y***			***confirm***	***y***	***y***	***y***	***n***
U373	***HTS QRT-PCR***	0,72	2,62	-0,93	0,05		U373	***HTS QRT-PCR***	0,67	1,62	-0,04	-0,5
	***QRT-PCR***	2,37	1,18	0,41	0,63			***QRT-PCR***	-1,98	0,05	1,25	-1,45
	***confirm***	***y***	***y***	***n***	***y***			***confirm***	***n***	***n***	***n***	***y***
												
	**miRNA-22**	**A**	**D**	**G**	**T**			**miRNA-145**	**A**	**D**	**G**	**T**
				
GBM2	***HTS QRT-PCR***	0,01	0,57	-3,53	-3,38		GBM2	***HTS QRT-PCR***	-5,7	-7,35	-2,72	-0,77
	***QRT-PCR***	-1,85	-0,87	-6,26	-2,16			***QRT-PCR***	-8,5	-1,5	-1,45	-1,64
	***confirm***	***n***	***n***	***y***	***y***			***confirm***	***y***	***y***	***y***	***y***
GBM5	***HTS QRT-PCR***	-1,08	-186	-2,07	-0,08		GBM5	***HTS QRT-PCR***	-2,19	-2,35	0,01	n.d.
	***QRT-PCR***	-2,15	-2,69	-3,18	-2,06			***QRT-PCR***	-4,75	-4,74	-2,1	-20,71
	***confirm***	***y***	***y***	***y***	***n***			***confirm***	***y***	***y***	***n***	
U373	***HTS QRT-PCR***	-2,52	-1,21	0,45	0,08		U373	***HTS QRT-PCR***	0,61	2,56	0,59	0,53
	***QRT-PCR***	-4,21	-4,1	-4,99	n.d.			***QRT-PCR***	1,61	1,54	0,5	1,47
	***confirm***	***y***	***y***	***n***				***confirm***	***y***	***y***	***y***	***y***

A: ALA; D: DHA; G: GLA; T: temozolomide

## 3.3 Validation of mRNA expression of selected miRNA target genes

Most of the targets of the differentially expressed miRNAs in response to PUFA treatment were apoptotic genes (Table 3). It is likely that the exhibited changes in miRNAs are as a consequence of apoptosis. This is supported by the fact that most of the mRNAs that are altered in response to PUFAs and temozolomide as noted in the present study are regulated by 11 miRNAs that can be classified as apoptosis-specific genes. Furthermore, most of the genes are overlapping (i.e. are targets for more than one miRNA), suggesting the existence of parallel regulatory pathways. These genes are the following: TP53INP1, MAP3K5, PDCD4, SIRT1, APP, BBC3, TNFRSF21, SGMS1.

**Table 3:** Genes having roles in apoptosis as targets of the differentially expressed miRNAs in response to PUFA treatment

MIRNA	GENE	Process	MIRNA	GENE	Process	MIRNA	GENE	Process
miR-17	APP	apoptosis	miR-30c	IL1A	apoptosis	miR-143	SMNDC1	apoptosis
	FASTK	apoptosis		DDIT4	apoptosis		NGFR	apoptosis
	EGLN3	apoptosis		SIRT1	apoptosis		KRAS	apoptosis
	SGMS1	apoptosis		TP53INP1	apoptosis		COX2	apoptosis
	TP53INP1	apoptosis		IRS2	apoptosis		BBC3	apoptosis
	TNFRSF21	apoptosis		ITGB3	apoptosis		ITM2B	apoptosis

miR-20b	APP	apoptosis	miR-16	CADM1	apoptosis	miR-22	DPF2	apoptosis
	EGLN3	apoptosis		BLC2	B cell proliferation		SIRT1	apoptosis
	E2F1	apoptosis		CADM1	cell adhesion		EP300	apoptosis
	FASTK	apoptosis		PDCD4	cell aging		YARS	apoptosis

miR-26a	DAPK1	apoptosis	miR-145	RTKN	apoptosis	miR-200a	PDCD1	apoptosis
	PAK2	apoptosis		PDCD4	apoptosis		NLRP3	apoptosis
	BAG4	apoptosis		UBE22	apoptosis		SGMS1	apoptosis
	UBE4B	apoptosis		IRS1	apoptosis		CTNNB1	apoptosis
	TP53INP1	apoptosis		RASSF5	apoptosis		NDUFS1	apoptosis

miR-29c	BBC3	apoptosis	miR-149	BBC3	apoptosis	miR-206	BDNF	anti-apoptosis
	SLK	apoptosis		PDE1B	apoptosis		API5	apoptosis
	AHR	apoptosis		PHF17	apoptosis		ZMAT3	apoptosis
	TP53INP1	apoptosis		TNFRSF19	apoptosis		BAG4	apoptosis
			
	ELMO2	apoptosis	miR-25	UBE22	apoptosis	miR-34	SIRT1	apoptosis
	RYBP	apoptosis		DYRK2	apoptosis		SGPP1	apoptosis
	PPARD	apoptosis		SGPP1	apoptosis			

**Figure 1:**
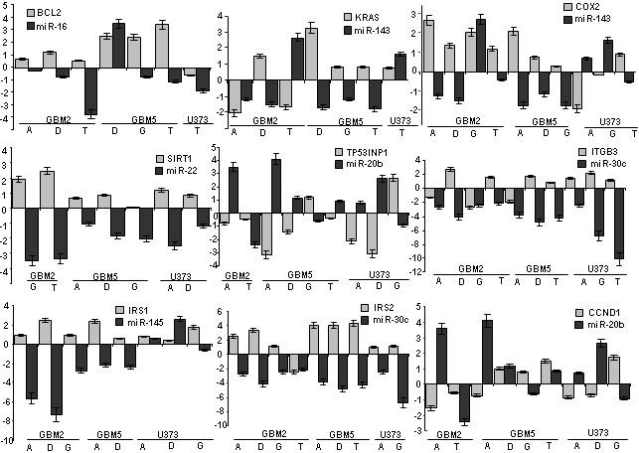
**Correlation between miRNA expression changes and alteration in mRNA level of different glioma cells after PUFA or temozolomide treatment**. „A" denotes for AA at 100 μM; „D" for DHA at 100 μM; "G" for GLA at 150 μM and „T" for temozolomide at 100 μM concentration.

To further validate our hypothesis we selected six miRNAs (mir-16, mir-20b, mir-22, mir-30c, mir-143 and mir-145) from the differentially expressed miRNAs and nine genes which are targets of the selected miRNAs. All the target genes code for proteins that have major role in triggering apoptosis in cancer cells. mRNA levels of target genes were determined by using gene-specific primers and traditional QRT-PCR. Total RNA was extracted from the same treated and control cells that was used for miRNA profling The expression of these mRNA coding genes were calculated (treated vs. un-treated) and the results are shown in Figure 1. It is evident that there is an inverse correlation between miRNA expression and mRNA expression changes as expected. In case of *bcl-2 *and *sirt-1 *this inverse correlation could be seen in 6 out of 7 cases when miRNA expression changes were recorded. Similar high correlation could be detected in case of *itgb3*, *k-ras*, *irs1*, *irs2*, cox2 and *ccnd1 *genes.

## Discussion

Understanding how the signaling pathways involved in surviving and inducing cell death of different glioma cells are regulated during PUFA treatment is important for the development of more effective tumor therapies including PUFAs alone or in combination with other drugs. In the present study, we focued on microRNA expression changes, as microRNAs are currently being recognized to have central role in the regulation of the expression of key gene families that are involved in cell physiology and the fate of cancer cells.

We have analyzed the expression of 112 microRNA by using the OpenArray™ nanocapillary HTS QRT-PCR technology (Applied Biosystems). This method merges the high-throughput of DNA-microarrays with the sound characteristics of QRT-PCR, therefore ideal for screening the expression of hundreds of genes from numerous samples. By using this technology and specific Taqman probes for each analyzed miRNA we generated raw expression data of three different glioblastoma cells (GBM2, GBM5 and U373) in response to different PUFA treatment. To confirm the utility of the HTS QRT-PCR method in miRNA expression analysis six miRNAs were selected and their relative expression ratios were determined in response to different treatments by using traditional QRT-PCR. 75% of the data corresponded well in both methods.

Relative changes in miRNA expression could be calculated for 65 miRNAs out of 112 with the HTS QRT-PCR method, because of the low or undetectable expression for the rest of the miRNA.

In order to differentiate specific responses to different PUFAs, we analyzed the effects of temozolomide, which is an oral alkylating agent that is used in the treatment of brain tumors [23]. The therapeutic benefit of temozolomide relies on its DNA methylation ability. This methylation damages the DNA and triggers the death of tumor cells at relatively high concentrations (over 100 μM) in culture media. We used temozolomide to identify miRNA expression changes that are due to apoptosis and to see whether there are differences or similarities between the changes caused by PUFAs.

In response to PUFA and temozolomide treatment, specific miRNA expression changes could be detected. However, in response to temozolomide we could record, in addition to specific miRNA changes, alterations in the expression of numerous other miRNAs that were similarly regulated regardless of the treatment. These common changes could be secondary to the induction of apoptosis by temozolomide and not as a result of specific mechanism of action of the molecule.

The miRNAs that showed such common changes were mir-34, mir-25, mir-17, mir-26a, mir-29c, mir-31, mir-200a and mir-206. However, we could detect temozolomide and PUFA-treatment specific alterations in miRNA expression. In case of temozolomide we found specific up-regulation of mir-182, and down-regulation of mir-16 and mir-183. Mir-143 was found to be repressed and mir-20b was induced by PUFA treatments.

It is interesting to note that different miRNAs were affected by treatment with the same PUFA in one cell line, but not in another, such as mir-125b, mir-197, mir-206. Similar cell-type specificity could be observed during general apoptosis, where all treatments (PUFAs and temozolomide) resulted in induced miRNA expression of mir-132, mir-140-3 and mir-323 in U373 cells. These differences can be attributed to the presence of different signalling pathways in different tumor cell lines, depsite the fact that the three studied cell lines were glioblastoma cells.

To confirm the importance of miRNA expression regulation during apoptosis, we listed the target genes of most of the miRNAs that were changed upon PUFA or temozolomide treatment. We found that most of the target genes can be functionally classified as apoptotic genes. Moreover, when we determined the expression of nine selected mRNA coding genes we found an inverse correlation between miRNA and mRNA expression in the same treated cells. The following genes were confirmed as regulated at the mRNA level and their expression was correlated to our miRNA expression data: *irs1, irs2, cox2, ccnd1, sirt1, tp53inp1, itgb3, k-ras *and *bcl2*.

In conclusion, we could demonstrate that in response to different PUFAs the expression of miRNA and the expression of their target mRNA coding genes were differentially altered. Most of the regulated genes could be classified as apoptotic genes and were up-regulated by PUFAs and temozolomide, while the same treatment resulted in repression of corresponding miRNAs. From these results, we conclude that PUFAs trigger apoptosis in glioma cells by regulating miRNA and their corresponding gene expressions.

## Conflict of interest

The authors declare that they have no competing interests.

## Authors' contributions

NF: carried out molecular genetic studies. LZF: carried out molecular genetic studies. KK: participated in the design of the study and performed the statistical analysis. UND: participated in the design and coordination of the study and drafted the manuscript. LGP: participated in the design and coordination of the study and drafted the manuscript.

All authors read and approved the final manuscript.

## Supplementary Material

Additional file 1 **QRT-PCR primers**. Primers used in this study. Click here for file

Additional file 2 **miRNA expression data**. Results of 112 analyzed miRNA expression in glioma cells (GBM2, GBM5, U373) in response to different PUFA (AA100, AA50, arachidonic acid at 100 and 50 μM concentrations, respectively; DH100, DH50, DHA at 100 and 50 μM concentrations, respectively; GL100, GL50, GLA at 100 and 50 μM concentrations, respectively) or temozolomide (TM100, 100 μM temozolomide) treatment. Click here for file
