# Supplementary material for: MicroRNA profile of polyunsaturated fatty acid treated glioma cells reveal apoptosis-specific expression changes
Source: Lipids Health Dis. 2011 Sep 30;10:173. doi: 10.1186/1476-511X-10-173 (PMC3203338; doi:10.1186/1476-511X-10-173)
Supplement: Additional file 1 — QRT-PCR primers. Primers used in this study. [file 1476-511X-10-173-S1.DOC]

**Additional Table 1**

| **Gene** | **Name** | **forward primer** | **reverse primer** |
| --- | --- | --- | --- |
| TP53INP1 | tumor protein p53 inducible nuclear protein 1 | aagactcacgggcacagaag | gcagcaagagctgcaacata |
| BCL2 | B-cell CLL/lymphoma 2 | tacctgaaccggcacctg | gccgtacagttccacaaagg |
| SIRT1 | sirtuin 1 | aaatgctggcctaatagagtgg | tggcaaaaacagatactgattacc |
| ITGB3 | integrin, beta 3 | ttcttcttggctctttaatgtaagc | cacaaatgctaaaagtccacagaa |
| IRS2 | insulin receptor substrate 2 | ttcttgtcccaccacttgaa | ctgacatgtgacatcctggtg |
| KRAS | v-Ki-ras2 oncogene | tggacgaatatgatccaacaat | tccctcattgcactgtactcc |
| COX2 | prostaglandin-endoperoxide synthase 2 | cttcacgcatcagtttttcaag | tcaccgtaaatatgatttaagtccac |
| CCND1 | cyclin D1 | gaagatcgtcgccacctg | gacctcctcctcgcacttct |
| IRS1 | insulin receptor substrate 1 | tatgccagcatcagtttcca | tttgctgaggtcatttaggtctt |

**Additional Table 2**

|  | **GBM2** | | | | | | | **GBM5** | | | | | | | **U373** | | | | | | |
| --- | --- | --- | --- | --- | --- | --- | --- | --- | --- | --- | --- | --- | --- | --- | --- | --- | --- | --- | --- | --- | --- |
| ASSAY | **AA100** | **AA50** | **DHA100** | **DHA50** | **GLA100** | **GLA50** | **TMZ100** | **AA100** | **AA50** | **DHA100** | **DHA50** | **GLA100** | **GLA50** | **TMZ100** | **AA100** | **AA50** | **DHA100** | **DHA50** | **GLA100** | **GLA50** | **TMZ100** |
| hsa-let-7a | **-0,27** | **0,06** | **-0,05** | **n.d.** | **0,15** | **0,80** | **-0,62** | **0,55** | **-0,30** | **-0,70** | **-0,45** | **-0,01** | **-0,18** | **-0,83** | **n.d.** | **n.d.** | **n.d.** | **n.d.** | **n.d.** | **n.d.** | **n.d.** |
| hsa-let-7b | **0,90** | **0,91** | **-0,74** | **-0,06** | **n.d.** | **n.d.** | **-8,60** | **n.d.** | **n.d.** | **n.d.** | **n.d.** | **-1,93** | **-0,84** | **-8,47** | **n.d.** | **-0,34** | **-1,57** | **-0,72** | **-2,54** | **-0,58** | **-1,21** |
| hsa-let-7c | **-0,36** | **-0,63** | **-0,58** | **-0,27** | **-1,02** | **-0,40** | **-0,93** | **-0,79** | **-0,74** | **-0,79** | **-0,49** | **-3,41** | **-2,37** | **-1,91** | **0,62** | **0,56** | **-0,49** | **-0,32** | **-1,86** | **-1,13** | **-0,99** |
| hsa-let-7d | **-0,39** | **-0,16** | **-0,57** | **-0,91** | **-0,25** | **-0,24** | **-0,37** | **1,99** | **1,83** | **1,73** | **2,00** | **-0,11** | **-0,57** | **0,52** | **2,08** | **1,91** | **-0,28** | **-0,17** | **0,06** | **-0,65** | **0,51** |
| hsa-let-7e | **1,04** | **0,98** | **-0,14** | **-0,34** | **1,33** | **1,55** | **0,97** | **1,33** | **2,10** | **1,30** | **1,25** | **-2,50** | **-2,75** | **-2,67** | **n.d.** | **n.d.** | **n.d.** | **n.d.** | **n.d.** | **n.d.** | **n.d.** |
| hsa-let-7f | **-0,79** | **0,11** | **0,32** | **-0,27** | **-0,25** | **0,53** | **-0,25** | **-2,57** | **-0,36** | **-1,34** | **-1,05** | **n.d.** | **n.d.** | **0,23** | **n.d.** | **n.d.** | **n.d.** | **n.d.** | **n.d.** | **n.d.** | **n.d.** |
| hsa-let-7g | **0,88** | **0,53** | **n.d.** | **n.d.** | **n.d.** | **n.d.** | **n.d.** | **0,42** | **0,49** | **-1,41** | **-1,62** | **0,08** | **-0,89** | **2,40** | **-4,72** | **-2,82** | **3,13** | **2,86** | **n.d.** | **n.d.** | **-6,09** |
| hsa-miR-1 | **-1,25** | **-1,17** | **-0,33** | **-0,84** | **-0,39** | **0,15** | **-0,46** | **-0,44** | **0,70** | **-0,48** | **-0,50** | **0,66** | **0,44** | **6,16** | **n.d.** | **n.d.** | **n.d.** | **n.d.** | **n.d.** | **n.d.** | **n.d.** |
| hsa-miR-100 | **n.d.** | **n.d.** | **n.d.** | **n.d.** | **n.d.** | **n.d.** | **n.d.** | **n.d.** | **n.d.** | **-2,56** | **-2,62** | **-1,65** | **n.d.** | **-0,72** | **n.d.** | **n.d.** | **n.d.** | **n.d.** | **-1,17** | **-2,04** | **n.d.** |
| hsa-miR-101 | **n.d.** | **n.d.** | **n.d.** | **n.d.** | **n.d.** | **n.d.** | **n.d.** | **n.d.** | **n.d.** | **n.d.** | **n.d.** | **n.d.** | **n.d.** | **n.d.** | **n.d.** | **n.d.** | **n.d.** | **2,34** | **n.d.** | **-2,75** | **-2,11** |
| hsa-miR-103 | **-0,68** | **-0,29** | **-1,29** | **-0,55** | **-0,78** | **-0,70** | **-1,00** | **n.d.** | **n.d.** | **n.d.** | **n.d.** | **n.d.** | **n.d.** | **n.d.** | **0,63** | **0,64** | **n.d.** | **1,57** | **-1,35** | **n.d.** | **-1,85** |
| hsa-miR-105 | **-0,68** | **-0,34** | **-0,08** | **-0,69** | **-0,23** | **0,00** | **-0,09** | **2,54** | **0,94** | **1,16** | **0,81** | **0,41** | **0,80** | **0,96** | **0,70** | **0,25** | **n.d.** | **n.d.** | **n.d.** | **n.d.** | **n.d.** |
| hsa-miR-106a | **0,17** | **-0,79** | **-1,93** | **-3,45** | **-0,90** | **-0,91** | **-0,59** | **-0,57** | **-0,48** | **0,05** | **0,11** | **n.d.** | **n.d.** | **-1,98** | **0,41** | **0,81** | **n.d.** | **-2,73** | **-3,22** | **n.d.** | **-3,97** |
| hsa-miR-106b | **-0,66** | **-0,99** | **-2,16** | **-2,00** | **-0,42** | **-0,44** | **-1,28** | **2,12** | **0,93** | **4,01** | **4,59** | **2,98** | **0,91** | **0,56** | **-0,38** | **-0,30** | **n.d.** | **n.d.** | **-4,91** | **n.d.** | **n.d.** |
| hsa-mi-R107 | **-0,30** | **-0,06** | **-0,73** | **-0,39** | **-0,14** | **-0,05** | **-0,56** | **0,16** | **0,91** | **2,30** | **2,95** | **n.d.** | **n.d.** | **n.d.** | **0,27** | **0,49** | **n.d.** | **n.d.** | **-1,92** | **n.d.** | **-2,43** |
| hsa-miR-10a | **-0,16** | **0,17** | **-0,59** | **-0,92** | **0,06** | **-0,06** | **-0,23** | **0,44** | **0,43** | **0,27** | **0,54** | **0,10** | **0,44** | **-0,40** | **0,81** | **0,37** | **-3,76** | **-0,39** | **-0,88** | **0,24** | **-1,94** |
| hsa-miR-10b | **-0,44** | **-0,15** | **-0,40** | **-0,75** | **-0,11** | **-0,07** | **-0,35** | **0,01** | **-0,62** | **-0,51** | **0,01** | **-1,93** | **-2,09** | **-0,36** | **n.d.** | **n.d.** | **0,21** | **0,25** | **3,05** | **0,50** | **-0,74** |
| hsa-miR-122 | **-0,70** | **1,00** | **0,80** | **-1,00** | **-0,09** | **-0,05** | **0,44** | **n.d.** | **n.d.** | **n.d.** | **n.d.** | **n.d.** | **n.d.** | **n.d.** | **n.d.** | **n.d.** | **n.d.** | **n.d.** | **n.d.** | **n.d.** | **n.d.** |
| hsa-miR-125b | **0,73** | **-0,01** | **0,31** | **0,67** | **n.d.** | **n.d.** | **n.d.** | **-4,51** | **-3,90** | **-4,48** | **-1,90** | **4,07** | **n.d.** | **-2,40** | **n.d.** | **n.d.** | **n.d.** | **n.d.** | **n.d.** | **n.d.** | **n.d.** |
| hsa-miR-126 | **-0,80** | **-0,21** | **-8,37** | **-4,99** | **-0,53** | **-0,13** | **-0,16** | **0,78** | **0,52** | **0,52** | **0,66** | **-0,66** | **0,52** | **-3,66** | **0,82** | **0,11** | **n.d.** | **n.d.** | **-1,86** | **n.d.** | **-1,16** |
| hsa-miR-127 | **-0,56** | **-0,76** | **n.d.** | **n.d.** | **-0,87** | **-0,66** | **-0,50** | **n.d.** | **n.d.** | **n.d.** | **n.d.** | **n.d.** | **n.d.** | **n.d.** | **n.d.** | **n.d.** | **n.d.** | **n.d.** | **n.d.** | **n.d.** | **n.d.** |
| hsa-miR-128a | **-1,22** | **0,35** | **-0,52** | **0,04** | **-0,18** | **-0,21** | **-0,30** | **0,96** | **0,57** | **0,76** | **0,55** | **-1,24** | **n.d.** | **-3,29** | **-0,40** | **-0,08** | **n.d.** | **-2,49** | **-3,99** | **n.d.** | **-1,87** |
| hsa-miR-129 | **n.d.** | **n.d.** | **n.d.** | **n.d.** | **n.d.** | **n.d.** | **n.d.** | **n.d.** | **n.d.** | **n.d.** | **n.d.** | **n.d.** | **n.d.** | **n.d.** | **0,53** | **-0,06** | **n.d.** | **n.d.** | **-8,75** | **n.d.** | **n.d.** |
| hsa-miR-130a | **-0,76** | **0,05** | **n.d.** | **n.d.** | **-0,31** | **-0,09** | **-0,56** | **n.d.** | **n.d.** | **n.d.** | **n.d.** | **n.d.** | **n.d.** | **n.d.** | **n.d.** | **n.d.** | **n.d.** | **n.d.** | **n.d.** | **n.d.** | **n.d.** |
| hsa-miR-130b | **-0,72** | **0,06** | **-2,47** | **n.d.** | **-0,66** | **-0,12** | **0,59** | **-5,21** | **-6,93** | **-5,31** | **-3,86** | **-0,97** | **-0,10** | **0,06** | **0,66** | **0,37** | **0,26** | **0,30** | **0,67** | **0,91** | **0,63** |
| hsa-miR-132 | **0,88** | **-0,06** | **n.d.** | **n.d.** | **-0,48** | **0,85** | **0,94** | **n.d.** | **n.d.** | **n.d.** | **n.d.** | **-0,73** | **-0,40** | **0,40** | **3,88** | **2,92** | **2,84** | **2,98** | **2,36** | **2,31** | **1,67** |
| hsa-miR-133a | **0,19** | **0,64** | **0,87** | **-0,37** | **-0,07** | **0,61** | **0,78** | **-0,23** | **-0,12** | **-0,32** | **0,36** | **0,15** | **-0,44** | **0,93** | **0,21** | **0,11** | **0,60** | **-0,03** | **-0,08** | **1,06** | **0,15** |
| hsa-miR-133b | **n.d.** | **n.d.** | **n.d.** | **n.d.** | **n.d.** | **n.d.** | **n.d.** | **n.d.** | **n.d.** | **n.d.** | **n.d.** | **n.d.** | **n.d.** | **0,32** | **0,81** | **0,67** | **0,07** | **-0,70** | **0,04** | **-0,62** | **2,02** |
| hsa-miR-135a | **n.d.** | **n.d.** | **1,40** | **n.d.** | **n.d.** | **n.d.** | **-1,65** | **n.d.** | **n.d.** | **n.d.** | **n.d.** | **n.d.** | **n.d.** | **n.d.** | **-0,68** | **0,52** | **-0,01** | **-0,43** | **n.d.** | **n.d.** | **0,51** |
| hsa-miR-136 | **n.d.** | **n.d.** | **n.d.** | **n.d.** | **n.d.** | **n.d.** | **n.d.** | **n.d.** | **n.d.** | **n.d.** | **n.d.** | **n.d.** | **n.d.** | **n.d.** | **n.d.** | **n.d.** | **n.d.** | **n.d.** | **n.d.** | **n.d.** | **n.d.** |
| hsa-miR-137 | **n.d.** | **n.d.** | **n.d.** | **n.d.** | **n.d.** | **n.d.** | **n.d.** | **n.d.** | **n.d.** | **n.d.** | **n.d.** | **n.d.** | **n.d.** | **n.d.** | **0,06** | **-0,39** | **-0,38** | **-0,51** | **0,10** | **-0,69** | **-0,78** |
| hsa-miR-138 | **2,23** | **-0,37** | **0,95** | **n.d.** | **-0,80** | **-0,31** | **0,72** | **n.d.** | **-2,06** | **n.d.** | **-4,24** | **-0,87** | **-0,85** | **-0,08** | **3,88** | **3,41** | **3,49** | **3,78** | **1,08** | **0,74** | **0,46** |
| hsa-miR-140-3p | **n.d.** | **n.d.** | **n.d.** | **n.d.** | **n.d.** | **n.d.** | **n.d.** | **n.d.** | **n.d.** | **n.d.** | **n.d.** | **n.d.** | **n.d.** | **n.d.** | **1,20** | **1,73** | **1,75** | **1,76** | **2,01** | **2,38** | **1,90** |
| hsa-miR-141 | **-0,57** | **-0,04** | **n.d.** | **n.d.** | **n.d.** | **n.d.** | **n.d.** | **0,24** | **-0,24** | **n.d.** | **n.d.** | **-0,65** | **-0,61** | **-1,13** | **0,42** | **0,12** | **3,68** | **3,00** | **1,98** | **2,72** | **1,25** |
| hsa-miR-143 | **-1,28** | **0,33** | **-1,52** | **-0,90** | **0,25** | **n.d.** | **-0,37** | **-1,76** | **-1,50** | **-1,19** | **-1,33** | **-1,78** | **-1,87** | **-0,14** | **0,68** | **0,73** | **1,63** | **2,71** | **-0,05** | **0,09** | **-0,50** |
| hsa-miR-145 | **-5,70** | **-4,30** | **-7,35** | **-2,37** | **-2,72** | **0,26** | **-0,77** | **-2,19** | **-2,82** | **-2,35** | **-1,35** | **0,01** | **-0,92** | **n.d.** | **0,61** | **0,14** | **2,57** | **2,43** | **0,59** | **1,60** | **0,53** |
| hsa-miR-146a | **n.d.** | **n.d.** | **-1,54** | **-0,86** | **-1,63** | **-0,01** | **-2,62** | **n.d.** | **n.d.** | **n.d.** | **n.d.** | **n.d.** | **n.d.** | **n.d.** | **n.d.** | **n.d.** | **n.d.** | **n.d.** | **n.d.** | **n.d.** | **n.d.** |
| hsa-miR-146b | **n.d.** | **n.d.** | **n.d.** | **n.d.** | **n.d.** | **n.d.** | **n.d.** | **-0,80** | **-0,95** | **-0,99** | **-0,98** | **0,40** | **-0,28** | **-0,98** | **0,24** | **0,45** | **0,69** | **0,49** | **-0,15** | **0,15** | **0,34** |
| hsa-miR-148a | **-0,40** | **-0,24** | **n.d.** | **n.d.** | **n.d.** | **n.d.** | **-0,52** | **n.d.** | **n.d.** | **n.d.** | **n.d.** | **n.d.** | **n.d.** | **n.d.** | **n.d.** | **n.d.** | **n.d.** | **n.d.** | **n.d.** | **n.d.** | **n.d.** |
| hsa-miR-149 | **0,47** | **-0,40** | **-0,06** | **-0,01** | **-0,87** | **-0,60** | **1,20** | **-3,84** | **-3,82** | **-2,15** | **-1,19** | **1,51** | **0,06** | **1,61** | **0,89** | **0,73** | **0,65** | **0,37** | **0,95** | **0,97** | **1,36** |
| hsa-miR-150 | **n.d.** | **n.d.** | **n.d.** | **n.d.** | **n.d.** | **n.d.** | **n.d.** | **n.d.** | **n.d.** | **n.d.** | **n.d.** | **n.d.** | **n.d.** | **-0,66** | **n.d.** | **n.d.** | **n.d.** | **n.d.** | **n.d.** | **n.d.** | **n.d.** |
| hsa-miR-152 | **n.d.** | **n.d.** | **n.d.** | **n.d.** | **n.d.** | **n.d.** | **n.d.** | **n.d.** | **n.d.** | **n.d.** | **n.d.** | **n.d.** | **n.d.** | **8,88** | **n.d.** | **n.d.** | **n.d.** | **n.d.** | **n.d.** | **n.d.** | **n.d.** |
| hsa-miR-153 | **0,47** | **-0,93** | **n.d.** | **n.d.** | **0,27** | **-0,40** | **n.d.** | **n.d.** | **n.d.** | **n.d.** | **n.d.** | **n.d.** | **n.d.** | **n.d.** | **n.d.** | **n.d.** | **n.d.** | **n.d.** | **n.d.** | **n.d.** | **n.d.** |
| hsa-miR-155 | **-0,53** | **-0,53** | **-0,72** | **-0,38** | **0,06** | **0,48** | **0,48** | **n.d.** | **n.d.** | **n.d.** | **n.d.** | **n.d.** | **n.d.** | **n.d.** | **n.d.** | **n.d.** | **n.d.** | **n.d.** | **n.d.** | **n.d.** | **n.d.** |
| hsa-miR-15a | **-0,17** | **-0,06** | **-1,13** | **-1,16** | **-0,73** | **-0,33** | **-0,56** | **0,09** | **0,97** | **0,25** | **0,27** | **-0,77** | **-5,55** | **-1,62** | **n.d.** | **n.d.** | **n.d.** | **n.d.** | **n.d.** | **n.d.** | **n.d.** |
| hsa-miR-15b | **-0,58** | **-0,17** | **-0,78** | **-0,64** | **-0,66** | **-0,19** | **-0,60** | **-0,40** | **0,01** | **-0,07** | **-0,06** | **-2,70** | **-3,90** | **-2,58** | **n.d.** | **n.d.** | **n.d.** | **n.d.** | **n.d.** | **n.d.** | **n.d.** |
| hsa-miR-16 | **-0,29** | **0,51** | **-0,78** | **0,22** | **n.d.** | **n.d.** | **-3,78** | **n.d.** | **n.d.** | **3,50** | **3,43** | **-0,75** | **n.d.** | **-1,13** | **n.d.** | **n.d.** | **-0,01** | **0,13** | **-0,15** | **0,22** | **-1,87** |
| hsa-miR-17 | **-1,29** | **-1,08** | **-0,87** | **-0,78** | **-1,34** | **-0,29** | **-1,28** | **0,01** | **0,72** | **0,80** | **0,58** | **-4,07** | **-4,96** | **-1,30** | **n.d.** | **n.d.** | **n.d.** | **n.d.** | **n.d.** | **n.d.** | **n.d.** |
| hsa-miR-181a | **n.d.** | **n.d.** | **n.d.** | **n.d.** | **1,83** | **0,75** | **-0,55** | **n.d.** | **n.d.** | **n.d.** | **n.d.** | **1,10** | **0,08** | **-0,93** | **1,50** | **-0,33** | **-0,38** | **0,55** | **3,59** | **3,00** | **-0,09** |
| hsa-miR-182 | **0,58** | **-0,80** | **-0,43** | **0,40** | **-0,31** | **0,01** | **2,42** | **-0,22** | **-0,09** | **-1,46** | **-1,62** | **-0,01** | **-0,60** | **1,06** | **0,70** | **0,47** | **0,81** | **0,65** | **0,40** | **0,21** | **0,23** |
| hsa-miR-183 | **0,32** | **-0,84** | **0,60** | **0,80** | **n.d.** | **4,02** | **-1,09** | **3,66** | **2,13** | **n.d.** | **n.d.** | **0,15** | **-0,22** | **-3,89** | **n.d.** | **n.d.** | **n.d.** | **n.d.** | **n.d.** | **n.d.** | **n.d.** |
| hsa-miR-184 | **-0,56** | **0,14** | **0,25** | **0,29** | **n.d.** | **4,04** | **-0,40** | **n.d.** | **n.d.** | **n.d.** | **n.d.** | **n.d.** | **n.d.** | **n.d.** | **n.d.** | **n.d.** | **-1,98** | **n.d.** | **n.d.** | **n.d.** | **n.d.** |
| hsa-miR-185 | **0,52** | **0,59** | **-0,50** | **0,18** | **-0,73** | **-0,73** | **0,04** | **-1,98** | **-0,89** | **-1,36** | **-0,31** | **0,92** | **0,57** | **0,03** | **0,92** | **0,78** | **0,39** | **0,58** | **0,45** | **0,65** | **0,33** |
| hsa-miR-18a | **-0,36** | **0,59** | **-0,23** | **-0,46** | **0,03** | **-0,27** | **-0,48** | **0,02** | **0,79** | **0,59** | **0,74** | **-1,03** | **-1,18** | **-1,28** | **n.d.** | **n.d.** | **n.d.** | **n.d.** | **n.d.** | **n.d.** | **n.d.** |
| hsa-miR-191 | **-0,27** | **0,83** | **1,31** | **-0,58** | **0,24** | **-0,43** | **1,28** | **-1,43** | **-1,36** | **-0,31** | **-0,15** | **1,00** | **-0,23** | **-2,38** | **n.d.** | **n.d.** | **n.d.** | **n.d.** | **n.d.** | **n.d.** | **n.d.** |
| hsa-miR-192 | **n.d.** | **n.d.** | **n.d.** | **n.d.** | **n.d.** | **n.d.** | **n.d.** | **n.d.** | **n.d.** | **n.d.** | **n.d.** | **n.d.** | **n.d.** | **n.d.** | **0,72** | **0,11** | **0,29** | **0,68** | **0,65** | **0,21** | **0,26** |
| hsa-miR-194 | **n.d.** | **n.d.** | **n.d.** | **n.d.** | **n.d.** | **n.d.** | **n.d.** | **n.d.** | **n.d.** | **n.d.** | **n.d.** | **n.d.** | **n.d.** | **n.d.** | **n.d.** | **n.d.** | **n.d.** | **n.d.** | **n.d.** | **n.d.** | **n.d.** |
| hsa-miR-196b | **0,15** | **-0,07** | **-0,78** | **-0,69** | **-0,42** | **0,13** | **-1,81** | **-0,14** | **0,77** | **-0,66** | **0,11** | **0,51** | **-0,46** | **-0,17** | **n.d.** | **n.d.** | **n.d.** | **n.d.** | **n.d.** | **n.d.** | **n.d.** |
| hsa-miR-197 | **-0,65** | **-0,83** | **-0,16** | **0,68** | **-0,88** | **0,43** | **-2,53** | **-0,10** | **-0,86** | **n.d.** | **n.d.** | **0,58** | **-0,88** | **0,85** | **0,01** | **0,78** | **2,07** | **1,42** | **2,57** | **2,67** | **-0,37** |
| hsa-miR-198 | **n.d.** | **n.d.** | **n.d.** | **n.d.** | **n.d.** | **n.d.** | **n.d.** | **n.d.** | **n.d.** | **n.d.** | **n.d.** | **0,03** | **-0,37** | **-1,69** | **-2,11** | **-1,80** | **-1,50** | **-1,61** | **-1,83** | **0,34** | **-5,82** |
| hsa-miR-199a | **0,14** | **-0,87** | **-0,92** | **-0,87** | **n.d.** | **5,22** | **1,62** | **n.d.** | **n.d.** | **n.d.** | **n.d.** | **2,60** | **-0,26** | **5,63** | **0,77** | **-0,69** | **0,25** | **0,91** | **1,48** | **0,02** | **-0,65** |
| hsa-miR-19a | **0,44** | **-0,17** | **-0,72** | **-0,65** | **0,37** | **0,72** | **0,82** | **n.d.** | **n.d.** | **n.d.** | **n.d.** | **-2,39** | **-0,73** | **-0,50** | **n.d.** | **n.d.** | **n.d.** | **n.d.** | **n.d.** | **n.d.** | **n.d.** |
| hsa-miR-19b | **n.d.** | **n.d.** | **n.d.** | **7,79** | **n.d.** | **n.d.** | **n.d.** | **n.d.** | **n.d.** | **n.d.** | **n.d.** | **1,47** | **0,68** | **-3,27** | **n.d.** | **n.d.** | **n.d.** | **7,38** | **-3,95** | **3,16** | **n.d.** |
| hsa-miR-200a | **3,40** | **3,62** | **n.d.** | **n.d.** | **9,66** | **8,88** | **0,78** | **n.d.** | **n.d.** | **n.d.** | **n.d.** | **0,53** | **0,98** | **4,64** | **-8,92** | **-3,53** | **-5,11** | **-7,75** | **-2,98** | **-5,51** | **-4,76** |
| hsa-miR-200c | **-3,74** | **-1,06** | **0,05** | **-0,12** | **1,28** | **-0,01** | **0,03** | **n.d.** | **n.d.** | **n.d.** | **n.d.** | **n.d.** | **n.d.** | **n.d.** | **n.d.** | **n.d.** | **n.d.** | **n.d.** | **n.d.** | **n.d.** | **n.d.** |
| hsa-miR-203 | **0,71** | **1,00** | **2,78** | **2,44** | **-18,98** | **-13,08** | **-4,79** | **n.d.** | **n.d.** | **n.d.** | **n.d.** | **n.d.** | **n.d.** | **n.d.** | **n.d.** | **n.d.** | **n.d.** | **n.d.** | **n.d.** | **n.d.** | **n.d.** |
| hsa-miR-204 | **n.d.** | **n.d.** | **n.d.** | **n.d.** | **n.d.** | **n.d.** | **n.d.** | **n.d.** | **n.d.** | **n.d.** | **n.d.** | **1,34** | **-0,01** | **0,10** | **n.d.** | **n.d.** | **n.d.** | **n.d.** | **n.d.** | **n.d.** | **n.d.** |
| hsa-miR-205 | **n.d.** | **n.d.** | **n.d.** | **n.d.** | **n.d.** | **n.d.** | **n.d.** | **n.d.** | **n.d.** | **n.d.** | **n.d.** | **n.d.** | **n.d.** | **n.d.** | **n.d.** | **n.d.** | **n.d.** | **n.d.** | **n.d.** | **n.d.** | **n.d.** |
| hsa-miR-206 | **0,39** | **-0,49** | **-2,29** | **-0,29** | **-3,46** | **-2,37** | **-1,22** | **n.d.** | **n.d.** | **n.d.** | **n.d.** | **2,25** | **1,41** | **2,20** | **n.d.** | **2,42** | **-2,86** | **0,73** | **0,75** | **-0,08** | **4,01** |
| hsa-miR-208 | **-1,97** | **-0,79** | **0,21** | **-0,55** | **-1,29** | **-2,44** | **-0,46** | **0,93** | **0,08** | **3,19** | **0,74** | **3,80** | **2,63** | **4,82** | **n.d.** | **1,08** | **n.d.** | **n.d.** | **n.d.** | **2,15** | **-0,06** |
| hsa-miR-20a | **0,76** | **0,59** | **-0,62** | **-0,33** | **-1,90** | **-1,60** | **-0,32** | **-0,56** | **0,26** | **-0,04** | **0,49** | **-3,25** | **-1,06** | **-3,06** | **n.d.** | **n.d.** | **n.d.** | **n.d.** | **n.d.** | **n.d.** | **n.d.** |
| hsa-miR-20b | **3,58** | **3,25** | **-0,41** | **0,40** | **n.d.** | **n.d.** | **-2,47** | **4,11** | **3,85** | **1,18** | **1,28** | **-0,64** | **0,53** | **0,86** | **0,73** | **-0,01** | **2,62** | **4,19** | **-0,94** | **0,20** | **0,06** |
| hsa-miR-21 | **n.d.** | **n.d.** | **n.d.** | **n.d.** | **n.d.** | **n.d.** | **n.d.** | **0,63** | **0,91** | **0,92** | **0,90** | **-2,02** | **-1,19** | **-0,27** | **n.d.** | **n.d.** | **n.d.** | **n.d.** | **n.d.** | **n.d.** | **n.d.** |
| hsa-miR-210 | **0,69** | **-0,29** | **-0,74** | **-0,43** | **-1,19** | **7,09** | **2,22** | **n.d.** | **-4,42** | **n.d.** | **n.d.** | **0,33** | **-0,33** | **0,85** | **0,67** | **-0,15** | **0,31** | **-0,20** | **0,83** | **0,78** | **0,17** |
| hsa-miR-214 | **n.d.** | **n.d.** | **-2,19** | **n.d.** | **-0,47** | **0,65** | **0,18** | **n.d.** | **n.d.** | **n.d.** | **n.d.** | **n.d.** | **n.d.** | **n.d.** | **0,91** | **0,93** | **0,56** | **0,98** | **0,07** | **0,02** | **0,81** |
| hsa-miR-219 | **0,08** | **0,42** | **n.d.** | **n.d.** | **1,74** | **0,78** | **-0,24** | **n.d.** | **n.d.** | **n.d.** | **n.d.** | **n.d.** | **n.d.** | **n.d.** | **n.d.** | **n.d.** | **n.d.** | **n.d.** | **n.d.** | **n.d.** | **n.d.** |
| hsa-miR-22 | **0,01** | **-0,16** | **0,57** | **-0,95** | **-3,54** | **-2,27** | **-3,39** | **-1,09** | **-1,17** | **-1,86** | **-0,66** | **-2,07** | **-1,01** | **-0,08** | **-2,52** | **-2,23** | **-1,22** | **-0,71** | **0,46** | **0,36** | **0,08** |
| hsa-miR-221 | **-0,39** | **-0,23** | **-0,07** | **-0,76** | **0,32** | **-0,30** | **0,08** | **3,79** | **1,34** | **n.d.** | **n.d.** | **n.d.** | **n.d.** | **0,21** | **n.d.** | **n.d.** | **n.d.** | **n.d.** | **n.d.** | **n.d.** | **n.d.** |
| hsa-miR-222 | **-0,32** | **0,41** | **-0,71** | **-0,95** | **2,23** | **2,16** | **0,90** | **-0,93** | **0,15** | **-0,77** | **0,35** | **n.d.** | **-2,30** | **n.d.** | **n.d.** | **n.d.** | **n.d.** | **-4,78** | **n.d.** | **-7,92** | **n.d.** |
| hsa-miR-223 | **n.d.** | **n.d.** | **n.d.** | **n.d.** | **n.d.** | **n.d.** | **n.d.** | **n.d.** | **n.d.** | **n.d.** | **n.d.** | **n.d.** | **n.d.** | **n.d.** | **n.d.** | **n.d.** | **n.d.** | **n.d.** | **n.d.** | **n.d.** | **n.d.** |
| hsa-miR-224 | **0,27** | **0,24** | **-0,92** | **-0,32** | **-1,47** | **-2,41** | **-0,97** | **n.d.** | **n.d.** | **n.d.** | **n.d.** | **n.d.** | **n.d.** | **n.d.** | **1,37** | **0,55** | **1,80** | **0,90** | **2,18** | **1,47** | **0,40** |
| hsa-miR-23a | **0,26** | **0,87** | **n.d.** | **n.d.** | **-0,59** | **-0,99** | **-2,23** | **-0,66** | **-0,21** | **0,06** | **-0,24** | **n.d.** | **n.d.** | **5,57** | **n.d.** | **n.d.** | **n.d.** | **n.d.** | **n.d.** | **n.d.** | **n.d.** |
| hsa-miR-23b | **-0,77** | **-0,59** | **-2,13** | **n.d.** | **-0,97** | **-0,40** | **-0,98** | **n.d.** | **n.d.** | **n.d.** | **n.d.** | **n.d.** | **n.d.** | **0,16** | **n.d.** | **n.d.** | **n.d.** | **n.d.** | **n.d.** | **n.d.** | **n.d.** |
| hsa-miR-24 | **3,26** | **3,18** | **n.d.** | **7,37** | **n.d.** | **n.d.** | **n.d.** | **n.d.** | **n.d.** | **-0,65** | **-0,64** | **0,75** | **0,16** | **0,72** | **n.d.** | **n.d.** | **n.d.** | **1,97** | **n.d.** | **-6,55** | **n.d.** |
| hsa-miR-25 | **-2,62** | **-1,15** | **-4,06** | **-1,40** | **-2,00** | **-1,22** | **-1,27** | **-1,33** | **0,09** | **-0,93** | **0,15** | **n.d.** | **n.d.** | **-3,33** | **n.d.** | **n.d.** | **n.d.** | **n.d.** | **n.d.** | **n.d.** | **n.d.** |
| hsa-miR-26a | **-0,87** | **-0,09** | **-0,77** | **-0,27** | **-0,36** | **0,32** | **-0,57** | **3,51** | **3,96** | **3,59** | **4,30** | **-0,84** | **0,28** | **-0,01** | **5,66** | **5,63** | **1,69** | **-0,42** | **3,17** | **n.d.** | **2,85** |
| hsa-miR-27a | **0,06** | **-0,07** | **0,42** | **0,58** | **-0,51** | **-0,50** | **-0,49** | **3,77** | **4,57** | **3,84** | **4,43** | **-1,00** | **-0,97** | **0,18** | **n.d.** | **n.d.** | **n.d.** | **n.d.** | **n.d.** | **n.d.** | **n.d.** |
| hsa-miR-27b | **-0,07** | **0,65** | **-0,47** | **-0,02** | **-0,11** | **0,05** | **-0,47** | **-0,18** | **0,39** | **0,82** | **1,22** | **n.d.** | **n.d.** | **0,24** | **n.d.** | **n.d.** | **n.d.** | **n.d.** | **n.d.** | **n.d.** | **n.d.** |
| hsa-miR-29a | **3,20** | **4,86** | **5,43** | **4,61** | **n.d.** | **n.d.** | **-5,47** | **n.d.** | **n.d.** | **1,38** | **0,47** | **0,24** | **0,69** | **-0,79** | **-2,64** | **0,43** | **n.d.** | **5,99** | **0,36** | **-0,79** | **-0,07** |
| hsa-miR-29b | **0,16** | **0,85** | **n.d.** | **n.d.** | **n.d.** | **n.d.** | **n.d.** | **n.d.** | **n.d.** | **-5,19** | **-6,51** | **n.d.** | **n.d.** | **0,12** | **n.d.** | **n.d.** | **n.d.** | **n.d.** | **n.d.** | **n.d.** | **n.d.** |
| hsa-miR-29c | **0,85** | **0,89** | **-4,50** | **-2,03** | **-1,75** | **-2,06** | **-2,41** | **n.d.** | **-0,14** | **-2,36** | **-1,82** | **-3,83** | **-1,45** | **-0,37** | **n.d.** | **n.d.** | **n.d.** | **n.d.** | **n.d.** | **n.d.** | **n.d.** |
| hsa-miR-30b | **0,10** | **-0,78** | **-1,89** | **-1,47** | **-0,53** | **-0,20** | **-0,56** | **0,00** | **0,36** | **0,11** | **0,74** | **n.d.** | **n.d.** | **-1,25** | **n.d.** | **n.d.** | **n.d.** | **n.d.** | **n.d.** | **n.d.** | **n.d.** |
| hsa-miR-30c | **-2,79** | **-2,92** | **-4,09** | **-4,93** | **-2,48** | **-2,07** | **-2,11** | **-3,88** | **-3,86** | **-4,82** | **-3,19** | **n.d.** | **n.d.** | **-4,27** | **-2,50** | **-4,70** | **n.d.** | **2,58** | **-6,85** | **n.d.** | **-10,13** |
| hsa-miR-31 | **n.d.** | **n.d.** | **-0,15** | **n.d.** | **n.d.** | **n.d.** | **n.d.** | **n.d.** | **n.d.** | **n.d.** | **n.d.** | **-0,16** | **0,00** | **0,46** | **-0,33** | **0,23** | **-4,69** | **n.d.** | **-4,13** | **-4,39** | **-4,23** |
| hsa-miR-32 | **n.d.** | **n.d.** | **n.d.** | **1,20** | **n.d.** | **n.d.** | **n.d.** | **n.d.** | **n.d.** | **n.d.** | **n.d.** | **-0,64** | **-0,49** | **2,55** | **n.d.** | **n.d.** | **n.d.** | **n.d.** | **n.d.** | **n.d.** | **n.d.** |
| hsa-miR-320 | **-0,65** | **0,02** | **-0,06** | **-0,65** | **0,39** | **0,03** | **-0,33** | **n.d.** | **-4,40** | **n.d.** | **n.d.** | **0,84** | **0,07** | **0,79** | **1,41** | **1,75** | **1,82** | **1,88** | **n.d.** | **n.d.** | **n.d.** |
| hsa-miR-323-3p | **n.d.** | **n.d.** | **n.d.** | **n.d.** | **n.d.** | **n.d.** | **n.d.** | **n.d.** | **n.d.** | **n.d.** | **n.d.** | **0,92** | **-0,38** | **-0,03** | **1,91** | **1,40** | **1,62** | **1,53** | **1,81** | **2,25** | **1,92** |
| hsa-miR-326 | **n.d.** | **n.d.** | **n.d.** | **n.d.** | **n.d.** | **n.d.** | **n.d.** | **n.d.** | **n.d.** | **n.d.** | **n.d.** | **-2,13** | **-0,09** | **-0,96** | **-3,04** | **-0,45** | **-2,88** | **0,04** | **-0,82** | **0,68** | **-1,52** |
| hsa-miR-328 | **n.d.** | **n.d.** | **n.d.** | **n.d.** | **n.d.** | **n.d.** | **n.d.** | **n.d.** | **n.d.** | **n.d.** | **n.d.** | **0,05** | **-0,15** | **0,29** | **0,98** | **0,24** | **0,95** | **0,42** | **1,95** | **2,37** | **-1,51** |
| hsa-miR-331 | **n.d.** | **n.d.** | **n.d.** | **n.d.** | **n.d.** | **n.d.** | **n.d.** | **n.d.** | **n.d.** | **n.d.** | **n.d.** | **-1,36** | **n.d.** | **-0,41** | **-0,08** | **-0,64** | **-0,42** | **-0,20** | **-0,64** | **0,98** | **0,19** |
| hsa-miR-335 | **n.d.** | **n.d.** | **n.d.** | **n.d.** | **n.d.** | **n.d.** | **n.d.** | **n.d.** | **n.d.** | **n.d.** | **n.d.** | **n.d.** | **n.d.** | **-0,55** | **0,83** | **0,10** | **-0,09** | **-0,45** | **1,12** | **0,62** | **-0,27** |
| hsa-miR-34a | **6,43** | **5,88** | **n.d.** | **n.d.** | **n.d.** | **n.d.** | **n.d.** | **0,60** | **0,00** | **0,03** | **0,45** | **-4,71** | **-3,80** | **-0,21** | **n.d.** | **n.d.** | **n.d.** | **n.d.** | **3,84** | **n.d.** | **n.d.** |
| hsa-miR-372 | **n.d.** | **n.d.** | **n.d.** | **n.d.** | **n.d.** | **n.d.** | **n.d.** | **n.d.** | **n.d.** | **n.d.** | **n.d.** | **n.d.** | **n.d.** | **n.d.** | **n.d.** | **n.d.** | **-0,34** | **-0,24** | **0,67** | **0,39** | **0,31** |
| hsa-miR-375 | **n.d.** | **n.d.** | **n.d.** | **n.d.** | **n.d.** | **n.d.** | **n.d.** | **n.d.** | **n.d.** | **n.d.** | **n.d.** | **n.d.** | **n.d.** | **n.d.** | **n.d.** | **n.d.** | **-0,63** | **-0,17** | **-0,53** | **-0,55** | **n.d.** |
| hsa-miR-382 | **n.d.** | **n.d.** | **n.d.** | **n.d.** | **n.d.** | **n.d.** | **n.d.** | **n.d.** | **n.d.** | **n.d.** | **n.d.** | **n.d.** | **n.d.** | **n.d.** | **n.d.** | **n.d.** | **n.d.** | **n.d.** | **0,95** | **-0,61** | **-0,45** |
| hsa-miR-451 | **n.d.** | **n.d.** | **n.d.** | **n.d.** | **n.d.** | **n.d.** | **-0,07** | **n.d.** | **n.d.** | **n.d.** | **-1,70** | **0,50** | **0,13** | **2,58** | **-0,29** | **-0,80** | **0,45** | **-0,68** | **-0,30** | **-0,32** | **-0,70** |
| hsa-miR-9 | **0,32** | **-0,84** | **-2,11** | **-1,04** | **-1,48** | **-0,44** | **-0,64** | **0,13** | **0,60** | **0,34** | **0,64** | **-2,70** | **-0,99** | **0,39** | **1,75** | **1,80** | **0,39** | **0,63** | **1,25** | **0,72** | **0,41** |
| hsa-miR-92a | **-0,45** | **-0,17** | **-0,71** | **-0,47** | **-0,27** | **-0,63** | **-0,64** | **2,47** | **2,00** | **2,33** | **2,49** | **-0,07** | **-0,99** | **0,31** | **1,34** | **1,07** | **n.d.** | **4,15** | **0,08** | **-0,86** | **-0,32** |
| hsa-miR-98 | **0,06** | **0,16** | **n.d.** | **n.d.** | **n.d.** | **n.d.** | **n.d.** | **n.d.** | **n.d.** | **n.d.** | **n.d.** | **n.d.** | **n.d.** | **n.d.** | **n.d.** | **n.d.** | **n.d.** | **n.d.** | **n.d.** | **n.d.** | **n.d.** |
| mmu-miR-124a | **n.d.** | **n.d.** | **n.d.** | **n.d.** | **n.d.** | **n.d.** | **n.d.** | **n.d.** | **n.d.** | **n.d.** | **n.d.** | **n.d.** | **n.d.** | **n.d.** | **n.d.** | **n.d.** | **n.d.** | **n.d.** | **n.d.** | **n.d.** | **n.d.** |
| mmu-miR-93 | **n.d.** | **n.d.** | **n.d.** | **n.d.** | **n.d.** | **n.d.** | **n.d.** | **n.d.** | **n.d.** | **n.d.** | **n.d.** | **n.d.** | **n.d.** | **n.d.** | **-1,84** | **-1,38** | **-3,75** | **-1,84** | **-2,96** | **-0,82** | **-2,06** |
| mmu-miR-96 | **0,38** | **-0,89** | **0,62** | **-0,72** | **0,69** | **0,05** | **-2,89** | **n.d.** | **-8,25** | **-0,25** | **0,08** | **-3,24** | **-3,21** | **-2,24** | **n.d.** | **n.d.** | **n.d.** | **n.d.** | **n.d.** | **n.d.** | **n.d.** |
